# Type of Explant Affects In Vitro Development and Multiplication Success of the Rare Halophyte Plant *Honckenya Peploides* L. Ehrh

**DOI:** 10.3390/plants9111526

**Published:** 2020-11-09

**Authors:** Danuta Kulpa, Mariola Wrobel, Martyna Bednarek

**Affiliations:** 1 Department of Plant Genetic, Breeding and Biotechnology, Faculty of Environmental Management and Agriculture, West Pomeranian University of Technology, Słowackiego 17, 71-434 Szczecin, Poland; martyna.bednarek@zut.edu.pl; 2 Department of Meteorology, Botany and Green Areas Management, Faculty of Environmental Management and Agriculture, West Pomeranian University of Technology, Słowackiego 17, 71-434 Szczecin, Poland; mariola.wrobel@zut.edu.pl

**Keywords:** micropropagation, psammophytes, halophytes, topophysis, salinity, position of explants, NaCl

## Abstract

The sea sandwort—*Honckenya peploides* (L.) Ehrh. is—a rare halophilous plant growing on dunes and is an endangered species on the Polish coast. It contributes to the stabilization of volatile sandy substrate, facilitating the colonization of other species. The present study determined the reaction of two types of explant: apical shoot fragments and fragments from a lower portion of the shoot. Apical shoot fragments were used to propagate and root sea sandwort plants due to the positive impact on the development of shoots and roots. Regardless of the plant growth regulators applied in the medium, the lateral meristems on the explants from the lower parts of the shoot stopped growing, and then yellowed and died out. Apical fragments of shoots developed higher and more numerous shoots and longer and more numerous roots than explants, which were fragments collected from lower parts of shoots. The findings indicated that propagation should be conducted on Murashige and Skoog medium with the addition of 1 mg∙dm^−3^ kinetin, whereas shoots with their apical fragments should be rooted with the addition of 1.5 mg∙dm^−3^ 1-naphthaleneacetic acid. The results also showed that the addition of NaCl at concentrations of 25 and 50 mM did not restrict their growth, thereby indicating the tolerance of the plant to soil salinity. However, an increase in the concentration of NaCl in the medium to 75 mM restricted the development of plants, and the shoots were lower and roots were shorter and less numerous.

## 1. Introduction

**Figure 1:**
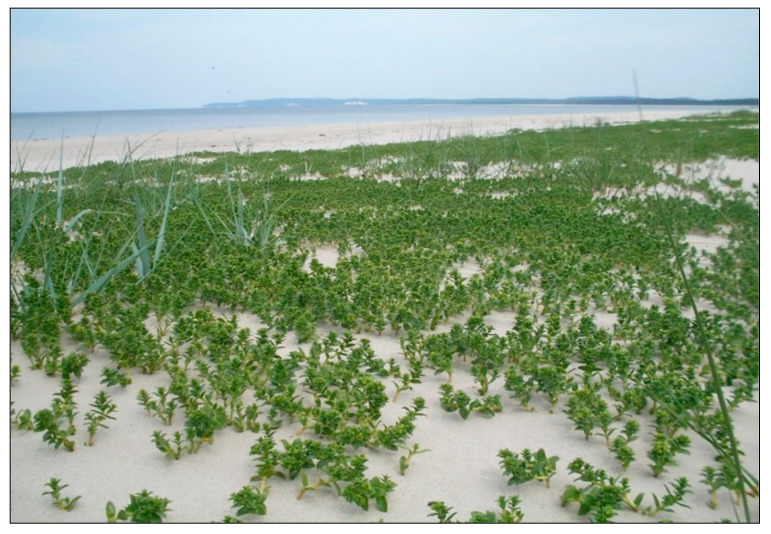
*Honckenya peploides* on the west coast of the Baltic Sea.

**Figure 2:**
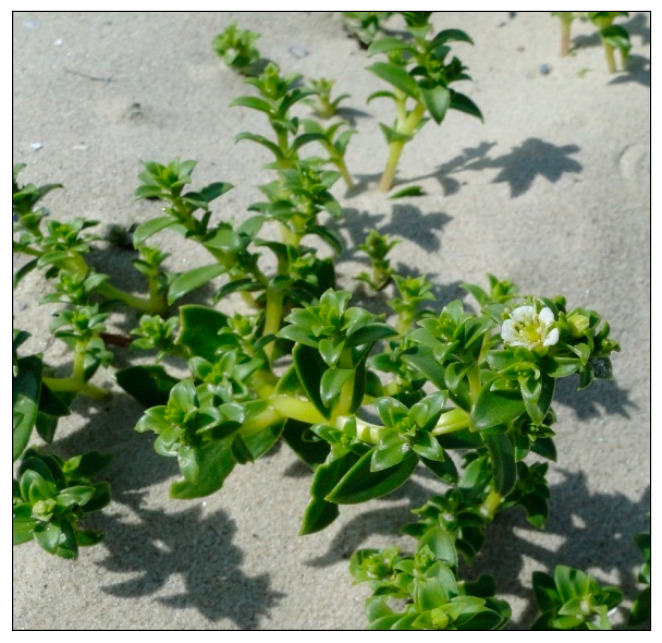
Flowering of *Honckenya peploides.*

Halophytes or halophilous plants are susceptible to the alterations of habitat conditions, and in particular, to reduced salinity and competition from other expansive species. The distribution of coastal halophytes is influenced by the regular supply of saline seawater during tides or high levels of stormwater [1,2]. Sea sandwort *Honckenya peploides* (L.) Ehrh. of the pink family *Caryophyllaceae* is an example of an obligatory halophyte requiring considerable soil salinity [3,4,5]. This plant has a subpolar distribution, stretching from the Arctic to the temperate zone in Western Europe, North America, and northwest Russia to Japan [6,7]. It occurs in the northern temperate zone between 30° and 80° latitude [6]. On coastal dunes, *H. peploides* colonizes the upper beach, where it forms small mounds known as embryo dunes, thereby stabilizing the volatile sandy substrate and facilitating colonization by other species (Figure 1 and Figure 2) [8,9,10,11]. It is the key species involved in the formation of permanent accumulative forms, which may transform into new dune ridges. In Poland, the sea sandwort has been designated as near-threatened and included in the Polish red list of ferns and flowering plants [12]. The existence of this rare plant in the Polish Baltic Sea coast is threatened by natural factors, such as storms and marine abrasion of beach habitats, and primarily affected by human activities, including mass tourism, recreational usage of sandy beaches, and transformation of dune habitats as well as development of the coast [13].

The sea sandwort is a utility plant in many countries. Juvenile sandwort shoots are consumed as vegetables, either in the raw or cooked form. They have a sour taste and a delicate flavor [14]. Sea sandwort plants have a high content of vitamins A and C. The leaves are pickled, and in Iceland, the entire plant is immersed in a sour whey and left to ferment. The alcoholic beverage thus obtained tastes similar to olive oil. The seeds, which are difficult to collect, are ground and added to flour. The Inuit people consume sandwort due to its medicinal properties. They collect the plant before flowering, in early summer. Juicy leaves and stems are cooked and consumed with seal oil [15].

Sea sandwort does not only propagate generatively but also vegetatively through buds on underground rootstock [5,16]. In vitro propagation is a method used to protect endangered species. It requires the precise selection of factors influencing the growth and development of plants under laboratory conditions, particularly the contents of plant growth regulators in medium to meet the needs of a given species. At present, the subject literature contains only a few studies on the possibility of in vitro propagation of halophytes and psammophytes owing to their long life cycles, heterozygosity, and difficulty in establishing in vitro cultures [17]. The problems associated with the reproduction of plants in this group include low efficiency of multiplication in in vitro cultures [18], high sensitivity to the effects of cytokinins usually added to the media during the multiplication stage in in vitro cultures, and frequent occurrence of hyperhydicity [19,20,21,22].

It was found that for several halophytes, NaCl addition to the medium in low concentrations (25 mM) promotes plant growth, both by increasing the number of leaves and improving the root system [23,24]. Other reports indicated that leaves and roots reacted differently to salt stress and were more sensitive to NaCl than the leaves. This may be explained by the hypothesis that the roots, which are the first part of a plant to encounter soil salinity, are more markedly affected by saline conditions than the leaves [25].

A critical stage of multiplication in in vitro cultures is rooting, which is particularly difficult in the case of halophytes. Very often, in the case of these, despite the high multiplication factor, the effectiveness of the culture is low, due to the small percentage of plants adapted to the in vivo conditions due to the poor development of the root system. During the rooting stage of many halophilic plants, auxins, especially 1-naphthaleneacetic acid (NAA), are added to the media [26,27]. Based on this, it can be assumed that the simultaneous application of an auxin and NaCl may have a beneficial effect on the rooting of *H. peploides* shoots. Determining the optimal NaCl content in the substrate, however, is not easy. In the studies by Ben Amour [23] on the microproduction of the halophilic species *Crithmum maritimum*, the increase in the above-ground and the underground parts was observed when plants were grown on a 50 mM medium. In the study of Joshi et al. [26] on the halophilic plant *Salicornia brachiata*, the salt content accumulating multiplication and rooting was much higher [250 and 500 mM].

Therefore, this study aimed to develop in vitro propagation methods for the sea sandwort (*H. peploides* (L.) Ehrh.). Particular attention was paid to the type of explants and the content of cytokinins, auxin NAA and NaCl in the medium.

## 2. Results

**Figure 3:**
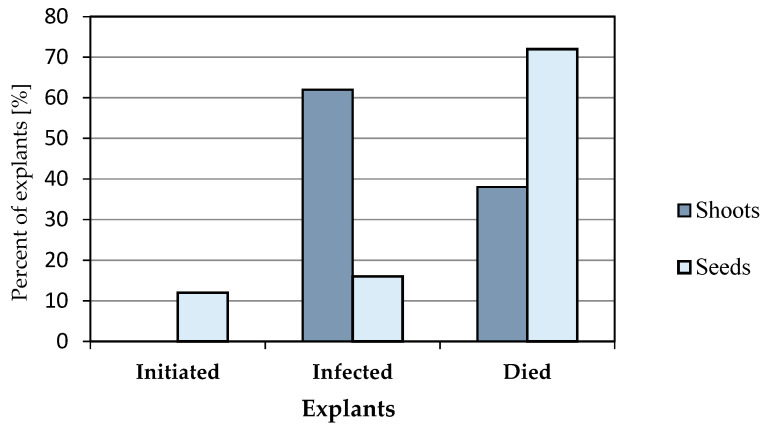
Percentage of explants [%] initiating growth, infected, and dead.

**Figure 4:**
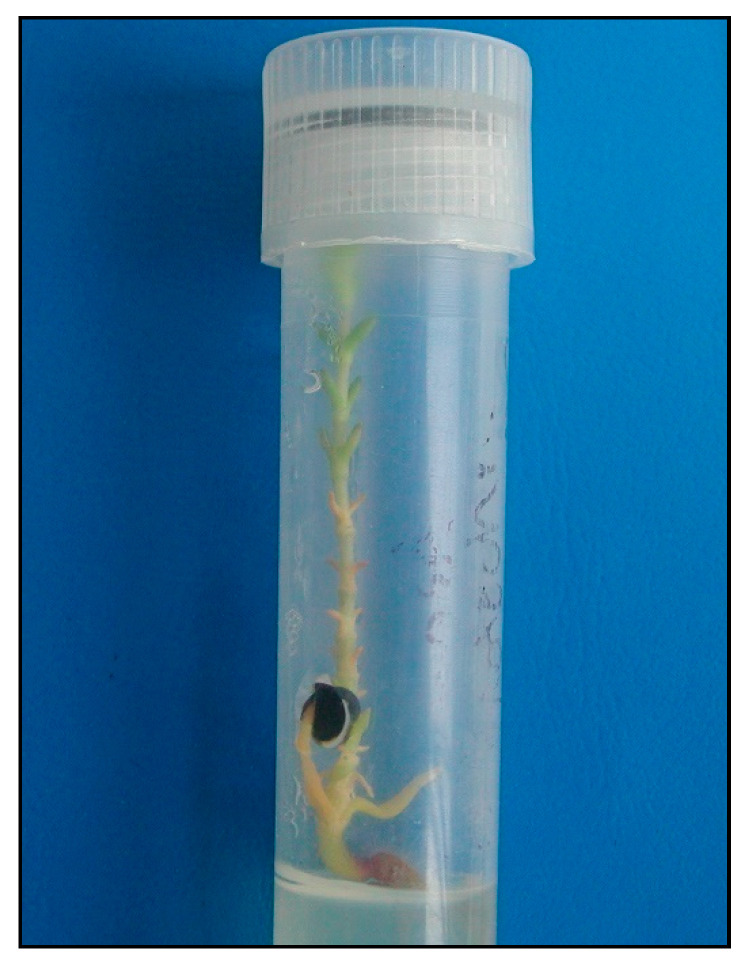
Micropropagation of *Honckenya peploides*: germinated seed.

*Initiation stage*. Two types of primary explants—shoot fragments and seeds—were introduced onto the media at the initiation stage. None of the shoot explants commenced growth, whereas 12% of the seeds placed on the medium were found to germinate (Figure 3 and Figure 4). The remaining seeds were infected and did not germinate.

**Figure 5:**
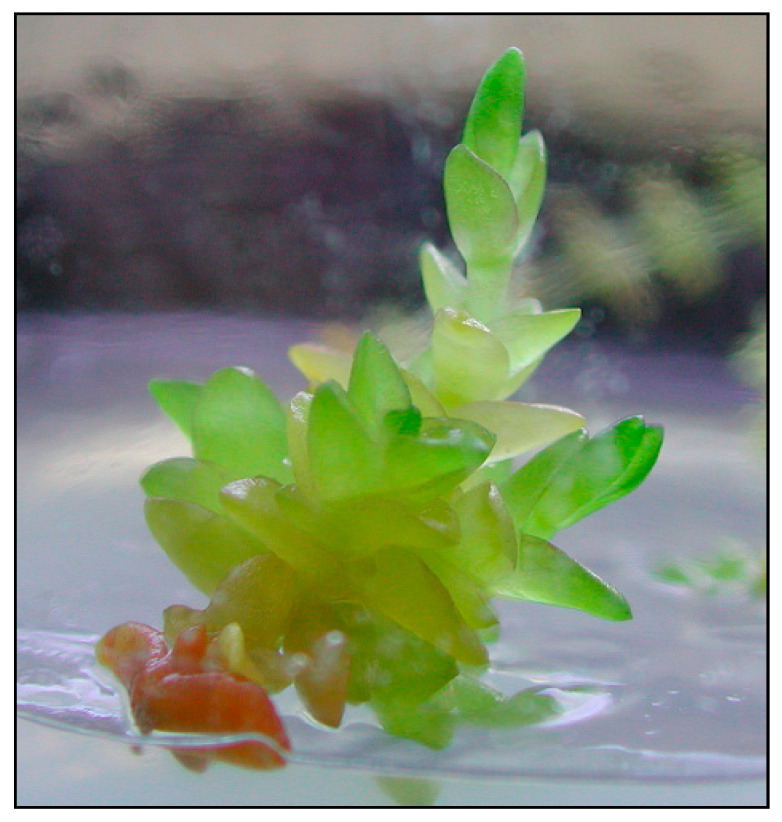
Top explant with symptoms of hyperhydicity regenerated on the Murashige and Skoog (MS) medium supplemented with 2 mg·dm^−3^ of 6-benzylaminopurine (BAP).

*Propagation stage.* At the propagation stage, the sea sandwort plants were characterized by a different appearance than that observed under natural conditions, regardless of the concentration and type of cytokinin added. The shoots were found to be yellowish, beginning from the plant base, indicating the symptoms of vitrification, particularly in the media containing the highest concentration of cytokinin (Figure 5).

The type and concentration of cytokinin added to the medium had a significant impact on the shoot length of sandwort (Table 1). Explants introduced in the media supplemented with kinetin (KIN) developed shoots with a height similar to plants propagated on the control medium (0.5 and 2 mg·dm^−3^) or were even taller than them (0.75 and 1 mg·dm^−3^). In turn, the remaining cytokinins (6-benzylaminopurine (BAP) and meta-topoline (mT)) added at any concentration resulted in decreased plant height. The lowest shoot height was observed in the sandwort plants propagated on the media supplemented with 1 and 2 mg·dm^−3^ mT and 0.5 mg·dm^−3^ BAP. It was also noted that the type of explant influenced the formation of shoot height. Shoots formed from the top fragments were almost twice as high (18.01 mm) as those formed from the shoot explants (9.99), wherein a strong growth inhibition was observed and some of the explants turned yellow and died.

**Figure 6:**
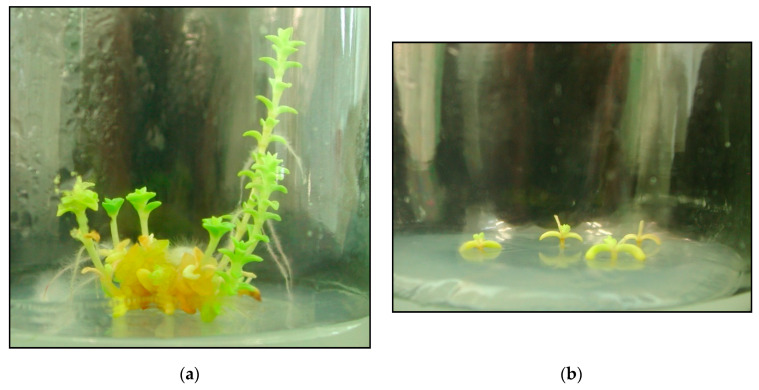
Top (**a**) and shoot (**b**) explant propagated on the MS medium supplemented with 1 mg·dm^−3^ of kinetin (KIN) after 6 weeks of growth.

The number of shoots formed was low, ranging between 1.00 and 2.00, with the exception of the sandwort propagated on media containing 1 mg·dm^−3^ KIN (from 1 to 7 shoots). A higher number of shoots than control was also observed in plants propagated on the medium containing 0.75 mg·dm^−3^ KIN and 1 mg·dm^−3^ mT. Apical explants developed more shoots (1.50) than shoot explants (1.11), with the highest number of shoots (4.33) seen in apical explants propagated on the medium added with 1 mg·dm^−3^ KIN (Figure 6a,b).

Cytokinins had a significant impact on the development of the root system. The longest roots, apart from the control medium, were developed by plants regenerated on media with the addition of mT (lengths between 5.25 and 11.75 mm). Explants introduced onto the media supplemented with 1 and 2 mg·dm^−3^ mT developed the highest number of roots (Table 1), whereas plants propagated on the medium supplemented with BAP and KIN at the highest concentrations did not develop a root system. Furthermore, the type of explant had a significant impact on the development of underground parts—the shoot fragments, in the majority of cases, did not develop a root system. Top explants did not develop a root system when they were placed on media with the highest concentrations of BAP and KIN. The length of explant roots originating from apical fragments was considerably higher than that of shoot explants (0.30 and 5.82 mm, respectively). Their count was also higher (0.15 for apical meristems and 0.99 for shoot fragments).

**Figure 7:**
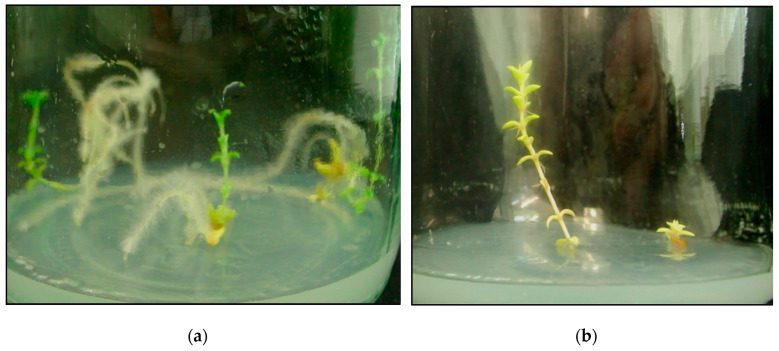
Rooting stage (6 weeks of growth). Top explants rooted on the medium with the addition of 1.5 mg·dm^−3^ NAA (**a**). Top (left) and shoot (right) explants rooted on the medium with the addition of 1 mg·dm^−3^ NAA (**b**).

*Rooting stage.* The concentration of NAA auxin (1-naphthaleneacetic acid) added to the medium and the type of explant used had a significant impact on the height of sandwort plants (Table 2). Sandwort shoots were smallest when they were rooted in the control medium (7.42 mm) but increased in height with the increase of NAA concentration. The average length of shoots developed from apical explants was fourfold greater than the shoot length observed in shoot explants (4.29 and 19.05 mm, respectively). Independent of the type, the explants showed the greatest shoot height, when they were rooted on the medium with 1.5 mg·dm^−3^ NAA (Figure 7a). However, a significant height difference was observed between shoot explants and apical fragments (7.69 and 26.10 mm, respectively). Regardless of the NAA concentration and explant type, sandwort plants developed one shoot.

Independent of the concentration, the addition of NAA stimulated rhizogenesis. The type of explant used also had a significant impact on the root length (Figure 7b). Apical explants developed a higher number (3.72) of roots, and the roots were longer (19.05). The highest number of roots (2.97 and 4.27, respectively) was developed by explants introduced onto media supplemented with 1 and 1.5 mg·dm^−3^ NAA, and the roots were also the longest (12.08 mm). Moreover, the interaction of the factors discussed turned out to be significant. The number of roots regenerated by apical explants was highest when the explants were introduced onto media supplemented with 1 and 1.5 mg·dm^−3^ NAA (4.13 and 6.17, respectively).

*Effects of NaCl*. Sea sandwort growing on media containing 25 and 50 mM NaCl developed shoots and root system similar to the control plants (Table 3). Increasing the soil salinity to 75 mM resulted in the development of the shortest shoots and shorter and less numerous roots. Independent of the NaCl content in the medium, sandwort plants developed a similar number of shoots (ranging from 1.00 to 1.14). Moreover, the plants developed from apical explants were taller and developed a higher number of longer roots than those developed from shoot explants.

## 3. Discussion

The present study determined the impact of cytokinins BAP, KIN, and mT on the propagation of plants, as well as NAA on the growth and rooting of the sea sandwort under in vitro cultures. The experiment also assessed the reaction of two types of explant—apical fragments and single-node fragments obtained from the lowest shoot portions—to the addition of plant growth regulators to media. In addition, the response of explants to the addition of NaCl to medium was examined.

It was observed that the type and concentration of cytokinin added to medium and the type of explant used had a significant impact on the length of sandwort shoots. The Murashige and Skoog (MS) medium containing KIN at the concentration of 1 mg·dm^−3^ was found to be the best for the propagation stage. The growth of the above-ground parts was strongly stimulated by this cytokinin, and apical explants developed as many as 4.33 shoots. KIN was also identified to be positively influencing the development of the above-ground part in the study of Kharrazi et al. [22] on *Dianthus caryophyllus*, another plant belonging to the pink family. It was observed that the addition of 1 mg·dm^−3^ KIN increased the length of shoots. Simona et al. [28] investigated the impact of growth regulators on *Dianthus chinensis*. When KIN was used at a concentration of 0.5–2.5 mg·dm^−3^, the plants developed the highest shoot; however, unfortunately, the highest percentage of vitrified plants was also obtained. In the present study, vitrification symptoms were observed in the population of plants propagated on media supplemented with the highest cytokinin concentrations.

The influence of the addition of 0.5, 1, and 1.5 mg∙dm^−3^ NAA (1-naphthaleneacetic acid) to the medium was determined in the subsequent study stage. It was found that the addition of the NAA auxin to medium stimulated rhizogenesis. The highest number of shoots was noticed on media added with 1 and 1.5 mg∙dm^−3^ NAA. Ali et al. [29] concluded that medium supplemented with 1 mg·dm^−3^ NAA is the best for in vitro rooting of *D. caryophyllus*.

In both study stages, a considerable difference was observed between the development of plants from apical explants and explants from the remaining portions of the shoot. The phenomenon of variable growth and development of seedlings based on their location in the mother plant is referred to as topophysis. The primary explant, which gives a start to a culture, transmits the memory of its status in the mother plant to the conditions of an in vitro culture. The study of Zalewska et al. [30] analyzed the impact of topophysical location of explants on the in vitro shoot regeneration efficacy in chrysanthemum. The authors examined the regeneration capacity of explants isolated from three topophysical zones: distal, central, and proximal. A sevenfold higher number of adventitious shoots were developed on the explants isolated from the distal proximal zone than from the central zone. Lu et al. [31] demonstrated that a higher number of adventitious shoots in chrysanthemum in vitro was formed from stem segments in the upper portions of shoots growing under in vivo conditions. In the present study, it was found that the explants isolated from the apical portion of shoots developed taller shoots, with longer and more numerous roots. On the other hand, the explants isolated from the lower portions of shoot attained a very small size, were yellowed, and withered. Such a large difference may stem from the adaptation of sandwort to inhabit dunes. During the stabilization of volatile sand and formation of the first aeolian dune forms, the sea sandwort exhibits high resistance to sand burying, and then it produces longer shoots that rise from underneath the layer of sand and the thick root system stabilizes the sand. The dune grows along with the growing sandwort patch. (Explants collected from there could have a higher growth rate.) Such shoots can be referred to as vegetative and are used for rapid colonization of new areas and formation of accumulative forms. The inflorescences appear only on older shoots and stabilized embryo dunes (generative shoots, and thus, the poorer growth rate of explants collected from flowering shoots). By expanding and attaining greater height over time, embryo dunes become an unfavorable place for sandwort; the limit dune height stabilized by sandwort is 0.5–1 m. Then, the sandwort individuals wither, and their underground shoots (rootstocks) and root system decompose, and they are replaced by new species (grasses) associated with ongoing succession. In a way, the sandwort is forced to colonize new beach surfaces in the direction of the sea [32].

In vitro propagation of halophilous plants is associated with several challenges. Addition of NaCl to medium was extremely critical for the propagation of another halophilous plant, *Salicornia brachiata,* under in vitro conditions by Joshi et al. [26]. In contrast to the study of Singh et al. [33] and Joshi et al. [34], in the present study the addition of NaCl to medium at concentrations of 25 and 50 mM neither stimulated the development of *Honckenya* nor had any negative impact on its growth, which may suggest the tolerance of the plant to soil salinity. However, increasing the soil salinity to 75 mM resulted in the development of the shortest shoots and shorter and less numerous roots.

## 4. Conclusions

This is the first study to report the methods of in vitro propagation of sea sandwort. The use of apical parts of shoots to propagate and root the sea sandwort (*H. peploides* (L.) Ehrh.) plants seems to be ideal with a positive impact on the development of shoots and roots. Propagation should be conducted on MS medium with the addition of 1 mg∙dm^−3^ KIN, whereas rooting should be performed with 1.5 mg∙dm^−3^ NAA. Addition of salt to medium does not have a positive impact on the in vitro development of *H. peploides*. The method developed can be used for propagating plants of such a high value for preserving a good state of the coastal environment.

## 5. Materials and Methods

### 5.1. Plant Material

**Figure 8:**
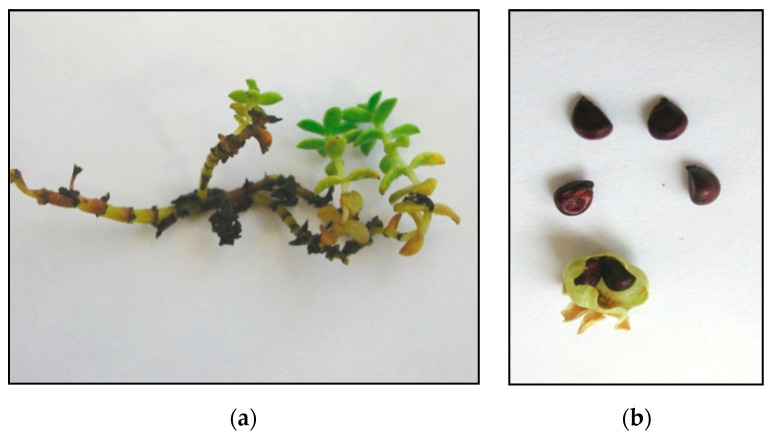
Starting material for tests: shoots (**a**) and seeds (**b**).

The starting materials for the experiment were seeds and 1-cm shoot fragments of the sea sandwort (*H. peploides* (L.) Ehrh.) (Figure 1, Figure 2 and Figure 8a,b) collected from the above-ground shoots growing on embryo dunes at the beach section between 423 and 422 km of the Polish Baltic Sea coast (N53.91609; E14.30809).

### 5.2. Culture Initiation and Stabilization Stage

The seeds and shoots were initially disinfected by rinsing in water containing a few drops of detergent. Subsequently, they were immersed in 70% ethanol for 30 s, and then in 10% sodium hypochlorite for 15 min, and were rinsed with sterile water three times. Explants were placed one by one into 10 mL test tubes, each containing 2 mL of MS medium [34]. After 6 weeks, the number of explants that initiated growth and were not infected were determined.

### 5.3. Propagation Stage

During this stage, as well as in the subsequent experimental stages, two types of explant were used: apical (top) shoot fragments measuring ~0.5 cm, containing apical meristem, and shoot fragments with the same length, collected from the bottom part of shoots with lateral meristems. The shoot fragments had one or two leaves. They were placed into jars containing MS media supplemented with cytokinins: BAP (6-benzylaminopurine), KIN (kinetin), or mT (meta-topoline)at a concentration of 0.5, 0.75, 1, and 2 mg⋅dm^−3^. Plants propagated on the MS medium without the addition of plant growth regulators constituted the control group.

### 5.4. Rooting Stage

The apical shoot fragments were used as explants and multiplied twice on the mineral medium according to Murashige and Skoog (1962) with the addition of 1 mg∙dm^−3^ KIN. Explants obtained from these 6-week-old plants were used to set up the next stage of the experiment. Two types of explant, as in the preceding study stages were placed on MS media supplemented with NAA at a concentration of 0.5, 1, and 1.5 mg⋅dm^−3^, respectively. Plants propagated on the MS medium without the addition of plant growth regulators constituted the control group.

### 5.5. Effect of NaCl

The starting material for the establishment of the experiment comprised 6-week-old plant shoots previously propagated on the MS medium supplemented with 1.5 mg⋅dm^−3^ NAA. Two types of explant were obtained from them, as in the preceding study stages, and were placed on the MS media supplemented with 1.5 mg⋅dm^−3^ NAA and NaCl at variable concentrations (25, 50, and 75 mM).

### 5.6. Culture Conditions

In all stages of the study, the media were supplemented with 30 g⋅dm^−3^ sucrose, 8 g⋅dm^−3^ agar, and 0.1 g⋅dm^−3^ inositol. Plant growth regulators were added to the medium before its pH was established at 5.7 ± 0.1 using 1 M solutions of HCl and NaOH. The test tubes and jars with media were sterilized in an autoclave at a temperature of 121 °C and a pressure of 0.1 hPa for 20 min. The cultures were performed in a growth chamber at 24 °C ± 1 °C, illuminated with a white fluorescent light having an intensity of 40 µEM^−2^s^−1^ PAR. A 16-h photoperiod and 8-h darkness cycle was applied. MS medium without the addition of plant growth regulators constituted the control group in each stage. During each experiment, apart from the culture initialization stage, the cultures were carried out in 330 mL jars containing 30 mL of medium. After 6 weeks, the height of plants (mm), number of developed lateral buds, and number and length of roots (mm) were determined.

### 5.7. Statistical Analysis

At the initiation stage, the experiment was conducted in a one-factor completely randomized design. It was performed in four replications with 50 explants each. In the next stages, the experiment was conducted in a two-factor completely randomized design. Each treatment at these stages consisted of 35 explants (seven replications of five explants each). An analysis of variance followed by Tukey’s test (*p* ≤ 0.05) was performed. The homogeneous groups between the examined combinations were labeled with successive letters of the alphabet. The percentage data were transformed to arc-sin before the analysis.

## Figures and Tables

**Table 1:** Effects of type explants and cytokinin content on the shoot multiplication of *Honckenya peploides.*

Cytokinin Content [mg·dm^−3^]		Plant Height [mm]			Number of Shoots			Roots Length [mm]			Number of Roots		
		Shoot	Top	Mean	Shoot	Top	Mean	Shoot	Top	Mean	Shoot	Top	Mean
**MS**	**0**	5.77	16.75	**11.26**	1.00	1.25	**1.13**	1.42	9.23	**5.32**	1.00	2.22	**1.61**
**BAP**	**0.50**	5.75	11.42	**8.59**	1.00	1.00	**1.00**	0	6.25	**3.13**	0	1.75	**0.88**
	**0.75**	6.25	21.00	**13.63**	1.25	1.25	**1.25**	0	5.00	**2.50**	0	0.75	**0.38**
	**1.00**	8.75	16.50	**12.63**	1.00	1.50	**1.25**	0	0	**0**	0	0	**0**
	**2.00**	7.25	11.50	**9.38**	1.00	1.25	**1.13**	0	0	**0**	0	0	**0**
**mT**	**0.50**	8.25	18.25	**13.25**	1.00	1.00	**1.00**	0	11.75	**5.88**	0	1.00	**0.50**
	**0.75**	12.00	19.50	**15.75**	1.00	1.00	**1.00**	0	11.50	**5.75**	0	0.50	**0.25**
	**1.00**	7.75	8.50	**8.13**	1.25	2.75	**2.00**	1.25	11.75	**6.50**	0.5	3.50	**2.00**
	**2.00**	6.42	6.50	**6.46**	1.00	1.25	**1.13**	1.25	9.25	**5.25**	0.5	1.50	**1.00**
**KIN**	**0.50**	9.75	26.00	**17.88**	1.00	1.75	**1.38**	0	5.50	**2.75**	0	0.75	**0.50**
	**0.75**	13.00	29.25	**21.13**	1.00	2.00	**1.50**	0	3.75	**1.88**	0	0.75	**0.38**
	**1.00**	12.00	30.00	**21.00**	1.75	4.33	**3.04**	0	1.67	**0.83**	0	0.17	**0.09**
	**2.00**	16.00	19.00	**17.50**	1.17	1.17	**1.17**	0	0	**0**	0	0	**0**
**Mean**		**9.99**	**18.01**		**1.11**	**1.65**		**0.30**	**5.82**		**0.15**	**0.99**	
LSD _0.05_		Media (M) = 1.63			Media (M) = 0.45			Media (M) = 2.06			Media (M) = 0.21		
		Explants (E) = 2.80			Explants (E) = 0.36			Explants (E) = 1.20			Explants (E) = 0.36		
		MxE = 2.31			MxE = non signifficant			MxE = 1.70			MxE = 0.29		
		ExM = 3.96			MxP = non signifficant			ExM = 2.91			ExM = 0.51		

**Table 2:** Effects of type explants and 1-naphthaleneacetic acid (NAA) content on the rooting of *Honckenya peploides.*

NAA Content [mg·dm^−3^]	Plant Height [mm]			Number of Shoots			Roots Length [mm]			Number of Roots		
	Shoot	Top	Mean	Shoot	Top	Mean	Shoot	Top	Mean	Shoot	Top	Mean
**0**	3.83	19.73	**11.78**	1.00	1.00	**1.00**	1.67	6.23	**3.95**	1.00	1.93	**1.47**
**0.5**	6.06	42.63	**24.34**	1.00	1.00	**1.00**	3.22	20.92	**12.07**	1.11	2.63	**1.87**
**1**	6.54	40.83	**23.69**	1.00	1.00	**1.00**	2.73	21.43	**12.08**	1.81	4.13	**2.97**
**1.5**	9.68	67.17	**38.43**	1.00	1.00	**1.00**	6.55	17.60	**12.08**	2.37	6.17	**4.27**
**Mean**	**6.53**	**42.59**		**1.00**	**1.00**		**3.54**	**16.55**		**1.57**	**3.72**	
LSD _0.05_												
**Media (M)**	1.18			non signifficant.			1.05			0.92		
**Explants (E)**	3.37			non signifficant			4.27			0.98		
**MxE**	1.42			non signifficant			1.23			0.85		
**ExM**	3.76			non signifficant			5.62			0.38		

**Table 3:** Effects of type of explant on the multiplication of *Honckenya peploides* propagated on MS media with the addition of 1.5 mg ·dm^−3^ NAA and varied NaCl content.

NaCl Content [mM]	Plant Height [mm]			Number of Shoots			Roots Length [mm]			Number of Roots		
	Shoot	Top	Mean	Shoot	Top	Mean	Shoot	Top	Mean	Shoot	Top	Mean
**0**	10.22	65.21	**37.72**	1.00	1.00	**1.00**	7.05	18.25	**12.65**	2.45	7.02	**4.74**
**25**	11.21	64.25	**37.73**	1.00	1.00	**1.00**	7.58	17.85	**12.72**	1.56	5.64	**3.60**
**50**	10.05	61.32	**35.69**	1.00	1.14	**1.07**	6.98	18.24	**12.61**	1.78	5.29	**3.54**
**75**	8.12	45.25	**26.69**	1.11	1.00	**1.06**	2.02	6.25	**4.14**	0.65	2.28	**1.47**
**Mean**	**9.90**	**59.01**		**1.03**	**1.05**		**5.91**	**15.15**		**1.61**	**5.06**	
LSD _0.05_												
**Media (M)**	5.23			non signifficant			2.36			1.32		
**Explants (E)**	2.35			non signifficant			1.06			0.56		
**MxE**	4.92			non signifficant			4.25			1.45		
**ExM**	3.25			non signifficant			2.56			0.82		

## Abbreviations

MS	Murashige and Skoog
NAA	1-naphthaleneacetic acid
BAP	6-benzylaminopurine
KIN	kinetin
mT	meta-topoline
